# Exercise Intensity-Dependent Effects on Cognitive Control Function during and after Acute Treadmill Running in Young Healthy Adults

**DOI:** 10.3389/fpsyg.2017.00406

**Published:** 2017-03-21

**Authors:** Martin Wohlwend, Alexander Olsen, Asta K. Håberg, Helen S. Palmer

**Affiliations:** ^1^Department of Circulation and Medical Imaging, Faculty of Medicine, Norwegian University of Science and TechnologyTrondheim, Norway; ^2^Department of Psychology, Faculty of Social Sciences and Technology Management, Norwegian University of Science and TechnologyTrondheim, Norway; ^3^Department of Physical Medicine and Rehabilitation, St. Olavs Hospital, Trondheim University HospitalTrondheim, Norway; ^4^Department of Neuroscience, Faculty of Medicine, Norwegian University of Science and TechnologyTrondheim, Norway

**Keywords:** physical activity, cognition, CCPT, reaction time, mood, PANAS

## Abstract

The idea that physical activity differentially impacts upon performance of various cognitive tasks has recently gained increased interest. However, our current knowledge about how cognition is altered by acute physical activity is incomplete. To measure how different intensity levels of physical activity affect cognition during and after 1 bout of physical activity, 30 healthy, young participants were randomized to perform a not-X continuous performance test (CPT) during low (LI)- and moderate intensity (MI) running. The same participants were subsequently randomized to perform the not-X CPT post LI, MI, and high intensity (HI) running. In addition, exercise related mood changes were assessed through a self-report measure pre and post running at LI, MI, and HI. Results showed worsening of performance accuracy on the not-X CPT during one bout of moderate compared to low intensity running. Post running, there was a linear decrease in reaction time with increasing running intensity and no change in accuracy or mood. The decreased reaction times post HI running recovered back to baseline within 20 min. We conclude that accuracy is acutely deteriorated during the most straining physical activity while a transient intensity-dependent enhancement of cognitive control function is present following physical activity.

## Introduction

Beside the well-established concept of primary and secondary prevention of lifestyle diseases through physical activity (Warburton et al., 2006), an emerging body of multidisciplinary research has linked physical activity to improvements in selective aspects of brain function and cognition (Booth and Lees, 2006; Vaynman and Gomez-Pinilla, 2006).

The most common measures of cognitive function in the exercise literature are measures of performance speed and accuracy. Evidence for an association between physical conditioning and improved reaction time was already reported in the early 20th century (Burpee and Stroll, 1936; Lawther, 1951; Pierson and Montoye, 1958). Since these early studies, a number of studies have investigated various variables in order to more specifically determine the impact of exercise on cognitive function, however, results have been somewhat conflicting (Tomporowski, 2003; Chang et al., 2012; McMorris and Hale, 2012). Reaction time changes seem to account for most of exercise’s observed effects on cognition (Tomporowski, 2003; McMorris and Hale, 2012), but the direction of this effect is unclear (Etnier et al., 1997; Chang et al., 2012; McMorris and Hale, 2012). Factors such as duration of the exercise intervention (acute vs. chronic exercise) (Hillman et al., 2008), timing of cognitive assessments (during vs. post-exercise) (Lambourne and Tomporowski, 2010; Chang et al., 2012; McMorris and Hale, 2012) and exercise modality (Lambourne and Tomporowski, 2010) appear to impact upon the results. The overwhelming majority of these studies have used static cycling as the exercise modality. Importantly, conflicting results can likely be attributed to poorly controlled exercise intensities (Lambourne and Tomporowski, 2010), which might be a major co-founding factor when comparing studies on exercise and cognition (Lambourne and Tomporowski, 2010). For cognitive tasks employing a measurement of information processing or executive function, results may additionally be conflicted by timing of test administration and exercise intensity (Lambourne and Tomporowski, 2010). Surprisingly, despite the rather large body of literature within exercise and cognition no study to date has compared the effect of controlled exercise intensities on cognitive function. Test administration during exercise has the largest effect on executive tasks, with impairments predominately up to 20 min (Lambourne and Tomporowski, 2010; Chang et al., 2012).

Despite conflicting results regarding reaction time in experimental exercise studies, various other cognitive processes have been shown to benefit from exercise. Particularly effects of exercise on cognitive control (executive function) processes seem to be disproportionately large (Hillman et al., 2008; Chang et al., 2012; McMorris and Hale, 2012). Cognitive control functions are those involved in goal-directed regulation of thoughts and emotions. This includes, but is not limited to, response inhibition, task-set maintenance, task switching, and mental flexibility, as well as other functions closely related to- or overlapping with attention and working memory. To extend findings from previous studies, it is crucial to both decipher the specific cognitive aspects amenable to exercise (i.e., reaction time and accuracy), and to optimize exercise protocols that aim to improve cognition and brain health. To this end, several variables have been studied and described to affect cognitive control function. Since exercise intensity is a major co-founding factor when trying to compare different studies, the current study sought to investigate intensity effects using a controlled, interventional design.

Cognitive control deficits are commonly observed in both neurological and psychiatric patient groups, including in anxiety- and mood disorders (Hammar and Ardal, 2009; Finnanger et al., 2013; Ryan et al., 2015; Shanmugan et al., 2016). The challenges presented by deficits in cognitive control can have a variety of negative consequences for quality of life across the lifespan and it is therefore desirable to investigate effective therapeutic interventions to address these deficits. Exercise has been shown to have beneficial effects on cognitive processes in younger adults (Chang et al., 2012; McMorris and Hale, 2012) and children (Sibley and Etnier, 2003). It is purported that exercise may also help to reduce the substantial age-related deterioration that has been shown in brain regions supporting cognitive control processes (chiefly the prefrontal cortex), (Hillman et al., 2008). Physical activity may indeed be beneficial for cognitive control throughout all stages of life but a greater understanding of the effects of exercise on cognitive control processes is needed in order to identify effective exercise intervention strategies.

Especially early exercise intervention as a cognitive intervention might be imperative for the maintenance of cognitive wellbeing and function throughout the adult lifespan (Hillman et al., 2008). Furthermore, several studies have investigated the effect of acute exercise on mood and a general improvement in mood after an acute single bout of exercise has been concluded (Yeung, 1996). However, it is more unclear how acute exercise affects positive and negative affection.

Given the prevalence of cognitive control deficits across different etiologies, and the fact that exercise may have a positive effect on such functions, gaining more detailed knowledge about this relationship has implications for the development of potential future interventions. The present study aimed to investigate the effect of different controlled exercise intensities on cognitive control function in healthy young adults as measured by reaction time and accuracy on a not-X continuous performance test (CPT) (Conners, 2000). Extending previous studies, a particularly rigorous randomized design with well-controlled exercise intensities was applied, and effects both during and after exercise were investigated. In addition, potentially associated mood alterations were explored.

## Materials and Methods

All subjects gave written informed consent prior to participation and the experimental procedures were in accordance with the Declaration of Helsinki. The experimental protocol was approved by the regional ethics committee (2011/1433).

### Experimental Design

**FIGURE 1:**
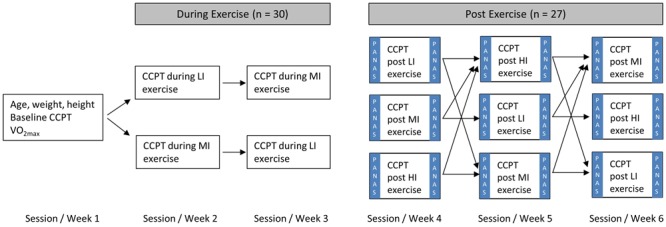
**Experimental protocol.** Cognitive function was assessed with a not-X continuous performance test (Conner’s Continuous Performance Test; CCPT). Low intensity (LI) exercise was defined as running at 40% maximal aerobic capacity (VO_2max_), moderate intensity (MI) as running at 60% VO_2max_ and high intensity (HI) as four intervals lasting 4 min each at 85% VO_2max_. The positive affection negative affection mood scale (PANAS) was used to investigate potential mood changes post-exercise.

The randomized multilevel cross-over experiment consisted of six sessions that took place over 6 weeks for each participant (see **Figure 1** for outline of experiment). The inter-session time interval was 1 week, and time of the day for testing was held constant (±1.5 h). In session 1, a baseline assessment of executive function represented by the participants’ reaction time and accuracy on Conners Continuous Performance Test [CCPT; 2nd ed.; CCPT-II Version 5 for Windows; (Conners, 2000)] was performed. Then the participants’ maximal aerobic capacity (VO_2max_) was tested. See below for detailed information about the CCPT and VO_2max_ test. During sessions 2 and 3, participants were randomized to either low intensity (LI) or moderate intensity (MI) exercise during performance of the CCPT. In sessions 4–6, participants performed CCPT after LI, MI, or high intensity (HI) exercise in a randomized order. In addition, they filled in a two-scaled mood test pre- and post LI, MI, or HI exercise. The randomization and the 1-week washout period between the sessions avoided task-learning effects as well as exercise-on task interference. Pilot testing showed that the CCPT task was too difficult to perform while running at HI, due to exacerbated arm and head movement. Therefore, we only compared CCPT performance during LI and MI running.

### Participants

Thirty (*n* = 30) healthy, young participants aged 18 to 35 (24.3 ± 3.3 years; 15 male, 15 female), were recruited from the local student population. Inclusion criteria were age (18–35) and a normal circadian rhythm, determined by interview, during the time course of the study. Exclusion criteria were history of heart conditions, neurological or psychiatric disorders. Participants were asked to refrain from caffeine use and physical activity, including a physically straining way to get to the laboratory on the test days. Two participants did not perform all post-exercise tests due to time constraints and personal reasons not related to the study, and were therefore excluded from the post-exercise test analyses. Another participant was excluded from the post-exercise test analyses on account of the CCPT results containing an unlikely high number of preservation and omission errors suggesting that this participant did not respond as instructed. Therefore, the during-exercise CCPT test data from all 30 participants were analyzed, but only 27 participants are included in the post-exercise CCPT test analyses and the analyses of pre- and post-exercise mood.

### Baseline Data

All testing was performed in the same environment in a training laboratory at St. Olavs Hospital in Trondheim, Norway. A treadmill (Technogym Runrace, Italy) was used for all treadmill running exercises. At the start of the experiment, weight was assessed with a digital weighing scale (Guangzhou Yimaijia Metal Products Co.), and height was measured using a simple wall mounted stadiometer (KWS Medical Supplies, Seattle, WA, USA). VO_2max_ measurements were obtained using the Oxycon Pro (Oxycon Pro, Erich Jaeger GmbH, Hoechberg, Germany) with a mouthpiece, which is connected to the volume transducer, together with a tube that collects samples of the gas concentration every 10 s. Prior to all VO_2_ measurements, the equipment was calibrated with a 3–1 standardized calibration syringe (Hans Rudolph Jäger GmbH, Germany), and the gas concentration sensor was calibrated with ambient air and a chemically standardized calibration gas with 16.0% O_2_, 4.0% CO_2_ and 80% nitrogen (SensorMedics Corporation, USA). Participants ran on the treadmill for 10 min at 7 km/h at 5% inclination to ensure that they were warmed-up, a large part of the blood volume was circulating and the muscles were warm to extract oxygen optimally. To obtain the VO_2max_ value, inclination was raised up to 10% in 0.5% increments every 0.5 min. If VO_2max_ was not achieved by that point, speed was increased by 1 km/h every min until achievement of VO_2max_. Criteria for achievement of VO_2max_ were a plateau in VO_2_ despite further increases in running speed or inclination, and a respiratory exchange ratio (R) of at least 1.08. For the measurement of heart rate, HR, Polar Accurex heart rate monitors (Polar Electro, Finland) were used. The highest HR during the last min of the VO_2max_ test was used as HR_max_. For all further training sessions, treadmill exercise workload was adjusted based on heart rate at a given %VO_2max_. The baseline data was not included in the statistical analyses but was used to characterize participants, familiarize them with the cognitive test and assess how the group worked cognitively.

### Exercise Intensities and Isocalorification of Energy Expenditure

Running exercise intensity was determined by %HR_max_. In the current study, LI exercise was defined as 40% VO_2max_ (∼63% HR_max_), MI as 60% VO_2max_ (∼75% HR_max_), and HI as 85% VO_2max_ (∼91% HR_max_). In sessions 2 and 3, participants warmed up for 5 min so that their %HR_max_ became stable at the correct intensity before starting the CCPT test whilst running. HI exercise consisted of 10 min warm-up at 40% VO_2max_ (∼63% HR_max_), followed by 4 min × 4 min interval session at 85% VO_2max_ with 3 min × 3 min recoveries at 40% VO_2max_ between each interval effort. The HI protocol ended with a 3 min cool-down session at 40% VO_2max_. To equate total work performed by the three exercise intensities for sessions 4–6, total oxygen cost for every individual was calculated from the HI protocol (22 min at 40% VO_2max_ and 16 min at 85% VO_2max_), similarly to what has been shown before (Rognmo et al., 2004):

Total oxygen cost of HI exercise =VO2max (L*min∧−1)*                                                                                            (22 min*0.4+16 min*0.85)

To calculate the running times at LI and MI with the same oxygen cost as the HI protocol:

$$\begin{array}{c} \mathrm{Time}(\min)\mathrm{at}LI=\frac{\mathrm{Total}\mathrm{oxygen}\mathrm{cost}\mathrm{of}\mathrm{HI}\mathrm{exercise}}{{\mathrm{VO}}_{2\max}(L*\min^{\wedge }-1)*0.4}\\ \mathrm{Time}(\min)\mathrm{at}MI=\frac{\mathrm{Total}\mathrm{oxygen}\mathrm{cost}\mathrm{of}\mathrm{HI}\mathrm{exercise}}{{\mathrm{VO}}_{2\max\;}(L*\min^{\wedge }-1)*0.6} \end{array}$$

### Assessment of Cognitive Control Function

Continuous performance test performance is subserved by brain regions involved in both transient and sustained cognitive control function (Olsen et al., 2013), and hypothesized to be particularly prone to the effects of exercise (Colcombe et al., 2004a,b). Furthermore, repeated testing of the CCPT-II showed less susceptibility to ceiling and floor effects (Reynolds and Kamphaus, 2003). Additonally, the duration of the CCPT (14 min) permits to investigate time-on-task effects. The CCPT-II was implemented to reliably assess both reaction time and accuracy related to cognitive control (McGee et al., 2000; Rabin et al., 2005; Strauss et al., 2007). Participants were instructed to click using a handheld USB Trackball mouse button (Digiflex, Wickford, UK) as soon as any letter from the alphabet except the letter “X” was displayed on a screen, and to withhold the response if the letter “X” was presented. A total of 324 targets (letters other than X) and 36 non-targets (the letter X) were presented in a pseudorandom fashion in six blocks. Interstimulus intervals (ISIs) varied between of 1, 2, and 4 s, and each single stimulus was displayed for 250 ms. Hit reaction time (Hit-RT), Hit-RT standard error of the mean (Hit-RT-SEM), and accuracy were calculated. Accuracy was defined as the sum of Omission and Commission errors, where Commission errors are hits on the non-target letter “X” and Omission errors are missed hits on target letters. A short practice test of 70 s was performed by all participants prior to the main test to familiarize the participants with the task. During the main test the examiner stayed in the room, but remained unobtrusive.

### Mood and Affection Scale

A short version of the positive affect negative affect scale (PANAS) was used to assess mood and affection (Watson et al., 1988). The scale consisted of two 10-item questionnaires, scale A and B. Each questionnaire has five items referring to positive affection and five items referring to negative affection. The short version, including its two scales, has been shown to be reliable, highly internally consistent and stable over a 2-month’s period (Watson et al., 1988). Significant changes in PANAS scores have been reported from 6 days or more (Bais et al., 2014) to as little as 10 min (MacKillop, 2006) in response to transmagnetic stimulation and cue exposure, respectively. Participants completed the PANAS mood scale A or B in a randomized order prior to exercise sessions 4–6, and completed the other version of the mood scale 3 min post-exercise.

### Statistical Analysis

Statistical analysis was performed using the software program SPSS 21.0 (IBM SPSS, New York, NY, USA). Paired-sample *t*-tests were conducted in order to assess differences in CCPT measures between MI and LI during exercise. The assumption of equality of variances was investigated using Leven’s test. Separate one-way repeated measures ANOVAs were conducted to investigate the effect of exercise intensity on each individual CCPT performance measure as well as positive affection and negative affection from pre- to post-exercise. The Mauchly’s test of Sphericity was used to investigate the assumption of equality of variances. Polynomial trend analyses were used to investigate the nature of significant Hit-RT over time effects between the exercise groups. All values are expressed as mean ± standard deviation (SD). A two-tailed *P* < 0.05 was accepted as statistically significant for all statistical tests.

## Results

### Participant Baseline Characteristics

Height, weight, age, maximal aerobic capacity (VO_2max_) and maximal heart rate (HR_max_) of the 30 participants (15 men) are shown in **Table 1**. As indicated by the VO_2max_ values, the participants in this study were well trained. At the time of testing all participants were students at the local university.

**Table 1:** Baseline data of the 30 participants (15 men).

Variable	Mean ±*SD*	*T*-score
Age (year)	24.27 @ 3.34	
Weight (kg)	70.9 @ 11.00	
VO_2max_ (ml^∗^kg^∗^min-1)	54.9 @ 7.70	
HR_max_ (min-1)	195 @ 6.50	
Hit reaction time (ms)	309.15 @ 39.42	35.62 @ 8.20
Accuracy	21.23 @ 9.52	47.77 @ 11.32
Reaction time standard error (ms)	4.19 @ 1.21	39.68 @ 8.25

*VO_*2max*_, maximal aerobic capacity; HR, heart rate; Accuracy, sum of Omission and Commission errors; *T*-score were calculated from the CCPT-II software. The *T*-score is inverted, lower scores are better.*

### CCPT Performance at Baseline, during, and Post-exercise

*Baseline*, CCPT performance measures and corresponding *T*-scores derived from norms available from the test developer are shown in **Table 1**. As indicated by the *T*-scores, the participants in this study can be considered a highly functioning group, particularly with respect to Hit-RT.

*During exercise*, participants were significantly more accurate during LI compared to MI [*t*(29) = -2.34, *p* = 0.026]. There was no difference in Hit-RT [t(29) = -0.22, *p* > 0.05] or Hit-RT-SEM [*t*(29) = -2.23, *p* > 0.05]. Results are presented in **Table 2**.

**Table 2:** Conners Continuous Performance Test (CCPT) performance during low-(LI) and moderate intensity (MI) running.

CCPT measure	LI	MI	*P-*value
**Hit reaction time (ms)**			
Mean ±*SD*	323.24 ± 34.11	324.07 ± 34.44	*p* > 0.05
**Accuracy**			
Mean ±*SD*	21.27 ± 10.91	24.43 ± 12.29^∗^	*p* = 0.026
**Reaction time standard error (ms)**			
Mean ±*SD*	3.64 ± 0.97	3.74 ± 1.05	*p* > 0.05

*Paired sample *t*-tests were used for statistical analyses. ^∗^Indicates *p* < 0.05.*

**FIGURE 2:**
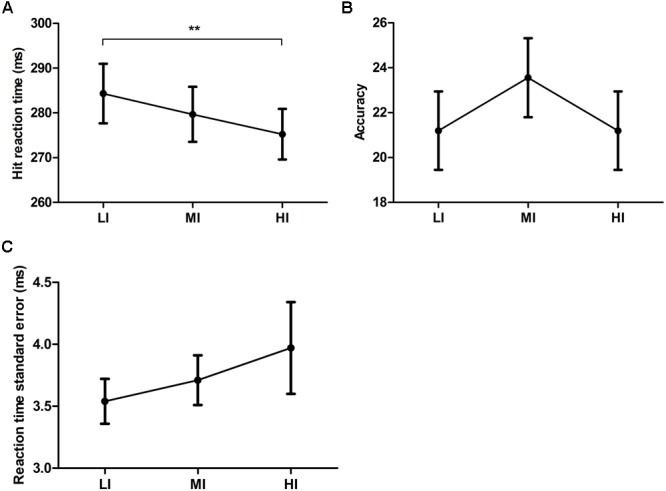
**Hit reaction time (A)**, accuracy **(B)** and reaction time standard error **(C)** post treadmill running exercise on low- (LI), moderate- (MI) and high- (HI) intensity. ^∗∗^Indicates *p* < 0.01. Error bars represent standard error mean.

*Post-exercise*, a significant main effect of exercise intensity for Hit-RT [*F*(2,52) = 4.47, *p* = 0.016, $\eta_{p}^{2}$ = 0.15] was present with a significant linear decrease in Hit-RT with increased exercise intensity [*F*(1,26) = 9.83, *p* = 0.0042, $\eta_{p}^{2}$ = 0.27] (**Figure 2A**). This improved performance did not come at the cost of accuracy [*F*(2,52) = 3.15, *p* > 0.05, $\eta_{p}^{2}$ = 0.11] or Hit-RT-SEM [*F*(1.26,32.86) = 0.99, *p* > 0.05, $\eta_{p}^{2}$ = 0.04] (**Figures 2B,C**).

**FIGURE 3:**
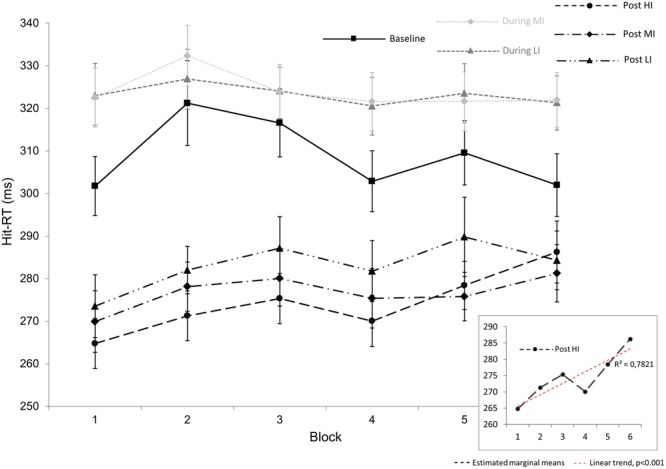
**Overview over the Hit reaction times (Hit-RT) during the 14 min of CCPT task performance.** On the *x*-axis, the 14 min of the CCPT task are summarized in six blocks, each lasting approximately 2 min and 20 s. Gray dotted lines depict Hit-RT during low- (LI) and moderate (MI) intensity exercise. Black dotted lines depict post-exercise Hit-RT during 14 min of CCPT task performance 5 min after LI, MI, and high- (HI) intensity. The black solid line represents changes in Hit-RT time during baseline condition. The lower right insert shows the significant linear nature of Hit-RT post HI exercise. The significant difference in Hit-RT post LI, MI, and HI exercise (average of all block values) is not shown here. Error bars represent standard error mean.

Follow-up analyses related to the significant effect for Hit-RT across exercise intensities were performed in order to investigate time on task effects (task block 1 through 6) during the post-exercise phase. There was a significant time on task effect for post HI exercise [*F*(5,130) = 8.45, *p* = 0.00000060, $\eta_{p}^{2}$ = 0.25]. A polynomial trend analysis revealed a linear increase in Hit-RT with time on task [*F*(1,26) = 33.63, *p* = 0.000004, $\eta_{p}^{2}$ = 0.27] in the post HI exercise period (insert **Figure 3**). There was no statistically significant time on task effects for LI post-exercise [*F*(3.06,79.5) = 2.61, *p* > 0.05, $\eta_{p}^{2}$ = 0.091] or MI post-exercise [*F*(3.56,92.45) = 2.42, *p* > 0.05, $\eta_{p}^{2}$ = 0.085]. Results are depicted in **Figure 3**.

### PANAS Mood Scale

Neither positive affection [*F*(2,52) = 1.85, $\eta_{p}^{2}$ = 0.066] nor negative affection [*F*(2,52) = 0.24, $\eta_{p}^{2}$ = 0.009] was affected by exercise intensity. Results are shown in **Table 3**.

**Table 3:** Positive and negative affection scores (PANAS) pre- and post low- (LI), moderate (MI), and high (HI) intensity running.

PANAS measure	LI	MI	HI
**Positive affection**			
Pre	13.44 ± 4.53	13.07 ± 3.75	13.52 ± 4.14
Post	13.78 ± 4.01	13.70 ± 4.31	15.22 ± 4.24
**Negative affection**			
Pre	7.11 ± 2.39	7.15 ± 2.51	7.07 ± 2.37
Post	6.62 ± 2.53	7.26 ± 4.55	6.96 ± 2.07

*One-way repeated measure ANOVAs were used to investigate whether exercise intensity had an effect on the change in either positive affection or negative affection from pre- to post-exercise. No significant differences (both ANOVAs *p* > 0.05) were found.*

## Discussion

The primary goal of the present study was to investigate the acute effect of running exercise intensity on cognitive control performance during and post treadmill running, as well as the effect of different exercise intensities on mood. The results from the study revealed three main findings with importance to understanding the acute effects of different exercise intensities on cognitive control function: (1) mean hit-RT in the post-exercise phase decreased linearly with increased exercise intensity, indicating that higher exercise intensity may be related to processes that facilitate neuronal efficiency, (2) hit-RT had a linear recovery to normal levels with time on task after HI exercise, indicating that the potential facilitating processes are transient, (3) changes in accuracy related to exercise intensity were only present during exercise, with participants demonstrating lower accuracy during MI intensity than LI exercise. There were no statistically significant effects of exercise intensity on accuracy post-exercise and no effects of exercise intensity on hit-RT during exercise. Moreover, this study did not show any statistically significant effects of exercise intensity on mood.

Our findings are in line with previous studies suggesting improved cognitive performance post-exercise (Lambourne and Tomporowski, 2010), and that such improvements might be linear with intensity (Chang et al., 2012). Similarly, the present study demonstrated a linear decrease in hit-RT with exercise intensity, resulting in a 3.2% improvement in hit-RT post HI compared to LI running. While previous studies comparing post-exercise with baseline point toward a decrease in reaction time immediately post running (McGlynn et al., 1979; Collardeau et al., 2001; Peiffer et al., 2015) and cycling (Hogervorst et al., 1996; Kashihara and Nakahara, 2005; Kamijo et al., 2007; Audiffren et al., 2008; Joyce et al., 2009), the effect of different intensities on reaction time post-exercise seems to be less clear, and the literature is comparatively sparse. No change in reaction time was observed in a flanker (Kamijo et al., 2007) and Go-NoGo task (Kamijo et al., 2004) post cycling at three different rates of perceived exhaustions. Similarly, a more recent study of elderly women found no difference in reaction time in a flanker task after 20 min of running at 50 and 75% VO_2max_ (Peiffer et al., 2015). In contrast, another study employing a visual search task indicated faster reaction times post 8 min at 65% plus 2 min at 85% workload, as compared to cycling 10 min at 65% workload (Aks, 1998). These contradictory findings can likely be attributed to poorly controlled energy expenditure and exercise intensity (Lambourne and Tomporowski, 2010).

Exercising at different intensities for the same amount of time results in different energy demands and oxygen usage, which might influence cognitive processing (Lambourne and Tomporowski, 2010). Moreover, determining exercise intensity by absolute workloads produces different levels of fatigue due to varying levels of cardiovascular fitness of participants. The present study used relative exercise intensities determined as %VO_2max_ making exercise intensities objectively and not subjectively comparable between participants. Moreover, running times for every participant were calculated so that exercise at all three intensities resulted in equal oxygen consumption. In summary, we found an exercise-intensity dependent decrease in hit-RT post one bout of exercise. There was no trade-off between hit-RT and accuracy, which suggests a linear improvement in cognitive processing with intensity post-exercise.

Exercise leads to undisputed health benefits including cognitive well-being. As early exercise intervention might be the future for cognitive wellbeing and function throughout the adult lifespan, optimization and identification of effective exercise strategies are needed. The present study identified exercise intensity as a potent modulator of cognitive control function with high-intensity-exercise being the strongest modulator.

The current results provide the first empirical evidence for a direct link between objective exercise intensity and reaction time in the post-exercise period. Participants had a mean *T*-score of 35.62 ± 8.20 (inverted) for reaction time at baseline (**Table 1**), performing on average about 1.5 standard deviations better than the norm group. Considering the fact that reaction times post-exercise still decreased argues for a rather strong biological effect. This acute effect happened despite the suggestion of a ceiling effect for exercise-related improvement to cognitive function during young adulthood (Hillman et al., 2008) as a result of a peak in cognitive function in young adults (Salthouse and Davis, 2006). Our findings are particularly interesting considering that simple reaction time has been reported to decline particularly after the age of 50, while choice reaction time declined linearly throughout life with 8% per decade (Der and Deary, 2006). Particularly stop signal reaction time deteriorated with 3% per decade, corresponding to 8–10 ms with every decade of aging (Smittenaar et al., 2015). Becoming 20–30 years younger in terms of reaction time post HI compared to LI might make an important difference in preventing falls as finger press reaction time has been shown to be a significant and independent predictor of falls (Lord et al., 1991, 1994; Lord and Clark, 1996). However, several factors should be further explored before drawing such conclusions.

By analyzing the time course of hit-RT throughout the entire 14 min of the not-X CPT test, we found that hit-RT post HI exercise climbs back toward baseline levels at the end of the test (**Figure 3**). This extends previous findings indicating that larger effects are observed after more intense exercise, with a peak about 11–20 min after exercise (Chang et al., 2012). In terms of simple reaction time, the strongest effect has been shown immediately after cessation of exercise (Collardeau et al., 2001) and similarly to our findings, another study found that the improvement in reaction time post running faded after a delay of 30 min (Peiffer et al., 2015).

The linear decrease in hit-RT after running might involve the norepinephrine and dopamine release from the adrenal medulla into the periphery, which increases with exercise intensity (Cooper, 1973; McMorris et al., 2008). This would suggest faster movement times post more intense exercise. However, it has previously been shown that movement time actually increased, slowing with higher intensity (Levitt and Gutin, 1971; Arent and Landers, 2003).

Central mechanisms involving increased excitability of the primary motor cortex post-exercise (unpublished transcranial magnetic stimulation data) may also play a role in the observed decrease in hit-RT post-exercise. This explanation is in line with the notion that exercise improves reaction time by energizing motor outputs (Audiffren et al., 2008).

Alternatively, or additionally, during exercise net influx of lactate from blood to brain might enhance bioenergetics of brain cells (Bergersen, 2015). As peripherally produced lactate is readily able to cross the blood–brain barrier and increases with exercise intensity, this mechanism might contribute to the differences in reaction time observed at increasing intensities. Another possible mechanism might be a regional shift in brain activity during exercise as a result of resource limitations as hypothesized by the reticular activated hypofrontality theory (Dietrich and Audiffren, 2011). Accordingly, a potential transient shift of brain resources to sustain intrinsic brain functions such as reaction time might be intensity-dependent. Future studies should go further into investigating underlying mechanisms and address how the acute improvements in reaction time post running from the present study translate into long-term adaptations.

In the present study, performance accuracy deteriorated during MI exercise compared to LI exercise whereas hit-RTs were highly similar (**Table 2**). This is in line with studies stating an overall negative effect on various cognitive tasks in healthy young adults during exercise (Lambourne and Tomporowski, 2010). Our finding of reduced accuracy during more intense exercise is in line with a study by McMorris et al. (2009) who found an increase in error rate at 80% compared to 50% *W*_max_ in a flanker task. Other cycling studies using the flanker task (Ando et al., 2011) or choice reaction time tasks (Delignieres and Brisswalter, 1994; Mouelhi Guizani et al., 2006) have not found altered accuracy at different exercise intensities. This may be due to the incremental protocols used or timing of testing (Chang et al., 2012). Achieving a stable heart rate (5 min) and completion of the CPT test (14 min) in the present study took around 19 min during running. Other studies indicate that positive acute effects of exercise on cognitive function peak between 15 and 20 min (Audiffren et al., 2008) whilst first impaired (Brisswalter et al., 2002; Lambourne and Tomporowski, 2010). There were no intensity-dependent alterations in hit-RT during exercise in our task, which is in line with several other studies in the field (Brisswalter et al., 1997; Mouelhi Guizani et al., 2006; Ando et al., 2011).

Attentional demands might be greater during running leaving less attentional resources for the cognitive task [dual-task effect; (Lambourne and Tomporowski, 2010)]. It has been suggested that the dual-task effect is strongly related to the energetic constraints of the task (Brisswalter et al., 2002). This could explain the increase in errors made during MI running compared to LI whereas when the immediate challenge of simultaneous running was not present post running, accuracy was not different between intensities and reaction time was positively affected. A recent study demonstrated that intensity of walking might affect dual task effects in a task-specific manner: a lower intensity of walking reduced the cognitive costs in the Stroop task but not in the visuomotor task while cognitive costs during walking at an increased intensity were highest for visuomotor reaction time tasks and lowest for the Stroop task (Patel et al., 2014). Hence, there might be an interaction of exercise intensity and dual-task effect, in addition to the type of task performed. In the present study, we found a deterioration in accuracy during MI compared to LI exercise but no change in hit-RT. These findings might be due to dual task effects and specific to exercise modality and timing of test administration.

A general improvement in mood after a single bout of exercise has been proposed (Yeung, 1996). Effects of exercise on mood were not observed in the current study. Other studies have found changes in mood using the PANAS mood scale after 4 weeks of exercise training, but not after one bout of exercise (Hopkins et al., 2012). This may indicate that the PANAS mood scale may not be sensitive enough to detect short-term changes in mood after only one session of exercise. Alternatively, short-term exercise as in the present study might not be enough to elicit an observable mood effect. However, reduced negative mood, as assessed by the Profile of Mood States (POMS), was reported post one exercise session at perceived intensities of 12–13 out of 20 (Fumoto et al., 2010). Another study employing POMS suggested an intensity-dependent decrease in vigor post acute exercise in an obese population (Lofrano-Prado et al., 2012). Hence, one bout of exercise seems to modulate mood in a positive manner and POMS might be a more sensitive test to detect such mood changes post short-term exercise. However, it remains unclear as to whether an acute bout of exercise has a robust, reproducible effect on mood.

A limitation to generalizing our findings is that we studied a well-functioning, healthy group of students. It has been argued that acute exercise effects are dependent on fitness level (Tomporowski, 2003; Chang et al., 2012). Moreover, gender-specific effects were not investigated. Considering that the effects of exercise may be rather subtle, there may be an elevated risk of type II errors. However, the randomized multilevel cross-over design was a strong feature of this study. Rather than being able to conclude effects on general cognition, the present study’s implications are limited to speed and accuracy during a cognitive control task, which arguably plays a crucial role for complex cognitive tasks including planning, abstract thoughts, cognitive flexibility or select relevant sensory information. Future studies should additionally investigate the effects of exercise intensity in other groups with a wider range of demographic characteristics, including patients with various conditions that may affect fitness level and/or cognition.

## Conclusion

In summary, the present study indicates that intensity might negatively affect cognitive control function during physical activity, and that a transient cognitive enhancement post one bout of exercise is intensity-dependent. Future studies should address long-term effects of physical activity at different intensities and identify mechanistic factors linking changes in neural activity with cognitive changes as a result of exercise.

## Author Contributions

MW: designed the study, performed experiments, analyzed data, and drafted manuscript. HP, AO, and AH: assisted in designing the study and edited the manuscript.

## Conflict of Interest Statement

The authors declare that the research was conducted in the absence of any commercial or financial relationships that could be construed as a potential conflict of interest.

## References

[B1] Aks D. J. (1998). Influence of exercise on visual search: implications for mediating cognitive mechanisms. *Percept. Mot. Skills* 87(3 Pt 1) 771–783. 10.2466/pms.1998.87.3.771 9885036

[B2] Ando S. Kokubu M. Yamada Y. Kimura M. (2011). Does cerebral oxygenation affect cognitive function during exercise? *Eur. J. Appl. Physiol.* 111 1973–1982. 10.1007/s00421-011-1827-1 21249389

[B3] Arent S. M. Landers D. M. (2003). Arousal, anxiety, and performance: a reexamination of the Inverted-U hypothesis. *Res. Q. Exerc. Sport* 74 436–444. 10.1080/02701367.2003.10609113 14768844

[B4] Audiffren M. Tomporowski P. D. Zagrodnik J. (2008). Acute aerobic exercise and information processing: energizing motor processes during a choice reaction time task. *Acta Psychol.* 129 410–419. 10.1016/j.actpsy.2008.09.006 18930445

[B5] Bais L. Vercammen A. Stewart R. van Es F. Visser B. Aleman A. (2014). Short and long term effects of left and bilateral repetitive transcranial magnetic stimulation in schizophrenia patients with auditory verbal hallucinations: a randomized controlled trial. *PLoS ONE* 9:e108828 10.1371/journal.pone.0108828 PMC4203691 25329799

[B6] Bergersen L. H. (2015). Lactate transport and signaling in the brain: potential therapeutic targets and roles in body-brain interaction. *J. Cereb. Blood Flow Metab.* 35 176–185. 10.1038/jcbfm.2014.206 25425080 PMC4426752

[B7] Booth F. W. Lees S. J. (2006). Physically active subjects should be the control group. *Med. Sci. Sports Exerc.* 38 405–406. 10.1249/01.mss.0000205117.11882.65 16540824

[B8] Brisswalter J. Arcelin R. Audiffren M. Delignieres D. (1997). Influence of physical exercise on simple reaction time: effect of physical fitness. *Percept. Mot. Skills* 85(3 Pt 1) 1019–1027. 10.2466/pms.1997.85.3.1019 9399313

[B9] Brisswalter J. Collardeau M. Rene A. (2002). Effects of acute physical exercise characteristics on cognitive performance. *Sports Med.* 32 555–566. 10.2165/00007256-200232090-00002 12096929

[B10] Burpee R. H. Stroll W. (1936). Measuring reaction time of athletes. *Res. Q. Am. Phys. Educ. Assoc.* 7 110–118. 10.1080/23267402.1936.10761762

[B11] Chang Y. K. Labban J. D. Gapin J. I. Etnier J. L. (2012). The effects of acute exercise on cognitive performance: a meta-analysis. *Brain Res.* 1453 87–101. 10.1016/j.brainres.2012.02.068 22480735

[B12] Colcombe S. J. Kramer A. F. Erickson K. I. Scalf P. McAuley E. Cohen N. J. (2004a). Cardiovascular fitness, cortical plasticity, and aging. *Proc. Natl. Acad. Sci. U.S.A.* 101 3316–3321. 10.1073/pnas.0400266101 14978288 PMC373255

[B13] Colcombe S. J. Kramer A. F. McAuley E. Erickson K. I. Scalf P. (2004b). Neurocognitive aging and cardiovascular fitness: recent findings and future directions. *J. Mol. Neurosci.* 24 9–14. 10.1385/jmn:24:1:009 15314244

[B14] Collardeau M. Brisswalter J. Audiffren M. (2001). Effects of a prolonged run on simple reaction time of well trained runners. *Percept. Mot. Skills* 93 679–689. 10.2466/pms.2001.93.3.679 11806586

[B15] Conners C. K. (2000). *CPT-II: Continuous Performance Test II: Computer Program for Windows Technical Guide and Software Manual.* Toronto, ON: Multi-Health Systems.

[B16] Cooper C. J. (1973). Anatomical and physiological mechanisms of arousal, with special reference to the effects of exercise. *Ergonomics* 16 601–609. 10.1080/00140137308924551 4772986

[B17] Delignieres D. Brisswalter J. (1994). Influence of an added perceptual motor task on perceived exertion: a test of the dissociation effect. *Percept. Mot. Skills* 78(3 Pt 1) 855–858. 10.2466/pms.1994.78.3.855 8084703

[B18] Der G. Deary I. J. (2006). Age and sex differences in reaction time in adulthood: results from the United Kingdom Health and Lifestyle Survey. *Psychol. Aging* 21 62–73. 10.1037/0882-7974.21.1.62 16594792

[B19] Dietrich A. Audiffren M. (2011). The reticular-activating hypofrontality (RAH) model of acute exercise. *Neurosci. Biobehav. Rev.* 35 1305–1325. 10.1016/j.neubiorev.2011.02.001 21315758

[B20] Etnier J. L. Salazar W. Landers D. M. Petruzzello S. J. Han M. Nowell P. (1997). The influence of physical fitness and exercise upon cognitive functioning: a meta-analysis. *J. Sport Exerc. Psychol.* 19 249–277. 10.1123/jsep.19.3.249

[B21] Finnanger T. G. Skandsen T. Andersson S. Lydersen S. Vik A. Indredavik M. (2013). Differentiated patterns of cognitive impairment 12 months after severe and moderate traumatic brain injury. *Brain Inj.* 27 1606–1616. 10.3109/02699052.2013.831127 24102501

[B22] Fumoto M. Oshima T. Kamiya K. Kikuchi H. Seki Y. Nakatani Y. (2010). Ventral prefrontal cortex and serotonergic system activation during pedaling exercise induces negative mood improvement and increased alpha band in EEG. *Behav. Brain Res.* 213 1–9. 10.1016/j.bbr.2010.04.017 20412817

[B23] Hammar A. Ardal G. (2009). Cognitive functioning in major depression–a summary. *Front. Hum. Neurosci.* 3:26 10.3389/neuro.09.026.2009 PMC2759342 19826496

[B24] Hillman C. H. Erickson K. I. Kramer A. F. (2008). Be smart, exercise your heart: exercise effects on brain and cognition. *Nat. Rev. Neurosci.* 9 58–65. 10.1038/nrn2298 18094706

[B25] Hogervorst E. Riedel W. Jeukendrup A. Jolles J. (1996). Cognitive performance after strenuous physical exercise. *Percept. Mot. Skills* 83 479–488. 10.2466/pms.1996.83.2.479 8902021

[B26] Hopkins M. E. Davis F. C. Vantieghem M. R. Whalen P. J. Bucci D. J. (2012). Differential effects of acute and regular physical exercise on cognition and affect. *Neuroscience* 215 59–68. 10.1016/j.neuroscience.2012.04.056 22554780 PMC3374855

[B27] Joyce J. Graydon J. McMorris T. Davranche K. (2009). The time course effect of moderate intensity exercise on response execution and response inhibition. *Brain Cogn.* 71 14–19. 10.1016/j.bandc.2009.03.004 19346049

[B28] Kamijo K. Nishihira Y. Hatta A. Kaneda T. Wasaka T. Kida T. (2004). Differential influences of exercise intensity on information processing in the central nervous system. *Eur. J. Appl. Physiol.* 92 305–311. 10.1007/s00421-004-1097-2 15083372

[B29] Kamijo K. Nishihira Y. Higashiura T. Kuroiwa K. (2007). The interactive effect of exercise intensity and task difficulty on human cognitive processing. *Int. J. Psychophysiol.* 65 114–121. 10.1016/j.ijpsycho.2007.04.001 17482699

[B30] Kashihara K. Nakahara Y. (2005). Short-term effect of physical exercise at lactate threshold on choice reaction time. *Percept. Mot. Skills* 100 275–291. 10.2466/pms.100.2.275-291 15974335

[B31] Lambourne K. Tomporowski P. (2010). The effect of exercise-induced arousal on cognitive task performance: a meta-regression analysis. *Brain Res.* 1341 12–24. 10.1016/j.brainres.2010.03.091 20381468

[B32] Lawther J. D. (1951). *Psychology of Coaching.* Oxford: Prentice-Hall, Inc.

[B33] Levitt S. Gutin B. (1971). Multiple choice reaction time and movement time during physical exertion. *Res. Q.* 42 405–410. 5291431

[B34] Lofrano-Prado M. C. Hill J. O. Silva H. J. Freitas C. R. Lopes-de-Souza S. Lins T. A. (2012). Acute effects of aerobic exercise on mood and hunger feelings in male obese adolescents: a crossover study. *Int. J. Behav. Nutr. Phys. Act* 9:38 10.1186/1479-5868-9-38 PMC3395553 22472267

[B35] Lord S. R. Clark R. D. (1996). Simple physiological and clinical tests for the accurate prediction of falling in older people. *Gerontology* 42 199–203. 10.1159/000213793 8832267

[B36] Lord S. R. Clark R. D. Webster I. W. (1991). Physiological factors associated with falls in an elderly population. *J. Am. Geriatr. Soc.* 39 1194–1200. 10.1111/j.1532-5415.1991.tb03574.x 1960365

[B37] Lord S. R. Ward J. A. Williams P. Anstey K. J. (1994). Physiological factors associated with falls in older community-dwelling women. *J. Am. Geriatr. Soc.* 42 1110–1117. 10.1111/j.1532-5415.1994.tb06218.x 7930338

[B38] MacKillop J. (2006). Factor structure of the alcohol urge questionnaire under neutral conditions and during a cue-elicited urge state. *Alcohol. Clin. Exp. Res.* 30 1315–1321. 10.1111/j.1530-0277.2006.00159.x 16899034

[B39] McGee R. A. Clark S. E. Symons D. K. (2000). Does the Conners’ continuous performance test aid in ADHD diagnosis? *J. Abnorm. Child Psychol.* 28 415–424. 10.1023/A:1005127504982 11100916

[B40] McGlynn G. H. Laughlin N. T. Rowe V. (1979). The effect of increasing levels of exercise on mental performance. *Ergonomics* 22 407–414. 10.1080/00140137908924625 477652

[B41] McMorris T. Collard K. Corbett J. Dicks M. Swain J. P. (2008). A test of the catecholamines hypothesis for an acute exercise-cognition interaction. *Pharmacol. Biochem. Behav.* 89 106–115. 10.1016/j.pbb.2007.11.007 18164752

[B42] McMorris T. Davranche K. Jones G. Hall B. Corbett J. Minter C. (2009). Acute incremental exercise, performance of a central executive task, and sympathoadrenal system and hypothalamic-pituitary-adrenal axis activity. *Int. J. Psychophysiol.* 73 334–340. 10.1016/j.ijpsycho.2009.05.004 19454298

[B43] McMorris T. Hale B. J. (2012). Differential effects of differing intensities of acute exercise on speed and accuracy of cognition: a meta-analytical investigation. *Brain Cogn.* 80 338–351. 10.1016/j.bandc.2012.09.001 23064033

[B44] Mouelhi Guizani S. Bouzaouach I. Tenenbaum G. Ben Kheder A. Feki Y. Bouaziz M. (2006). Simple and choice reaction times under varying levels of physical load in high skilled fencers. *J. Sports Med. Phys. Fitness* 46 344–351. 16823368

[B45] Olsen A. Ferenc Brunner J. Evensen K. A. Garzon B. Landro N. I. Haberg A. K. (2013). The functional topography and temporal dynamics of overlapping and distinct brain activations for adaptive task control and stable task-set maintenance during performance of an fMRI-adapted clinical continuous performance test. *J. Cogn. Neurosci.* 25 903–919. 10.1162/jocn_a_00358 23363414

[B46] Patel P. Lamar M. Bhatt T. (2014). Effect of type of cognitive task and walking speed on cognitive-motor interference during dual-task walking. *Neuroscience* 260 140–148. 10.1016/j.neuroscience.2013.12.016 24345478

[B47] Peiffer R. Darby L. A. Fullenkamp A. Morgan A. L. (2015). Effects of acute aerobic exercise on executive function in older women. *J. Sports Sci. Med.* 14 574–583. 26336345 PMC4541122

[B48] Pierson W. R. Montoye H. J. (1958). Movement time, reaction time, and age. *J. Gerontol.* 13 418–421. 10.1093/geronj/13.4.418 13611237

[B49] Rabin L. A. Barr W. B. Burton L. A. (2005). Assessment practices of clinical neuropsychologists in the United States and Canada: a survey of INS, NAN, and APA Division 40 members. *Arch. Clin. Neuropsychol.* 20 33–65. 10.1016/j.acn.2004.02.005 15620813

[B50] Reynolds C. R. Kamphaus R. W. (2003). “Handbook of psychological & educational assessment of children,” in *Personality, Behavior, and Context* 2nd Edn eds Reynolds C. R. Kamphaus R. W. (New York, NY: The Guilford Press) 718.

[B51] Rognmo O. Hetland E. Helgerud J. Hoff J. Slordahl S. A. (2004). High intensity aerobic interval exercise is superior to moderate intensity exercise for increasing aerobic capacity in patients with coronary artery disease. *Eur. J. Cardiovasc. Prev. Rehabil.* 11 216–222. 10.1097/01.hjr.0000131677.96762.0c 15179103

[B52] Ryan K. A. Dawson E. L. Kassel M. T. Weldon A. L. Marshall D. F. Meyers K. K. (2015). Shared dimensions of performance and activation dysfunction in cognitive control in females with mood disorders. *Brain* 138(Pt 5) 1424–1434. 10.1093/brain/awv070 25818869 PMC4840457

[B53] Salthouse T. A. Davis H. P. (2006). Organization of cognitive abilities and neuropsychological variables across the lifespan. *Dev. Rev.* 26 31–54. 10.1016/j.dr.2005.09.001

[B54] Shanmugan S. Wolf D. H. Calkins M. E. Moore T. M. Ruparel K. Hopson R. D. (2016). Common and dissociable mechanisms of executive system dysfunction across psychiatric disorders in youth. *Am. J. Psychiatry* 173 517–526. 10.1176/appi.ajp.2015.15060725 26806874 PMC4886342

[B55] Sibley B. A. Etnier J. L. (2003). The relationship between physical activity and cognition in children: a meta-analysis. *Pediatr. Exerc. Sci.* 15 13 10.1123/pes.15.3.243

[B56] Smittenaar P. Rutledge R. B. Zeidman P. Adams R. A. Brown H. Lewis G. (2015). Proactive and reactive response inhibition across the lifespan. *PLoS ONE* 10:e0140383 10.1371/journal.pone.0140383 PMC4619547 26488166

[B57] Strauss E. Sherman E. M. S. Spreen O. (2007). A compendium of neuropsychological tests: administration, norms and commentary. *Appl. Neuropsychol.* 14 62–63. 10.1080/09084280701280502

[B58] Tomporowski P. D. (2003). Effects of acute bouts of exercise on cognition. *Acta Psychol.* 112 297–324. 10.1016/S0001-6918(02)00134-8 12595152

[B59] Vaynman S. Gomez-Pinilla F. (2006). Revenge of the “sit”: how lifestyle impacts neuronal and cognitive health through molecular systems that interface energy metabolism with neuronal plasticity. *J. Neurosci. Res.* 84 699–715. 10.1002/jnr.20979 16862541

[B60] Warburton D. E. Nicol C. W. Bredin S. S. (2006). Health benefits of physical activity: the evidence. *CMAJ* 174 801–809. 10.1503/cmaj.051351 16534088 PMC1402378

[B61] Watson D. Clark L. A. Tellegen A. (1988). Development and validation of brief measures of positive and negative affect: the PANAS scales. *J. Pers. Soc. Psychol.* 54 1063–1070. 10.1037/0022-3514.54.6.1063 3397865

[B62] Yeung R. R. (1996). The acute effects of exercise on mood state. *J. Psychosom. Res.* 40 123–141. 10.1016/0022-3999(95)00554-4 8778396

